# Effect of Genipin Crosslinking on the Cellular Delivery of Gelatin Nanocarriers Using Curcumin as a Payload

**DOI:** 10.1155/ijbm/9512576

**Published:** 2026-02-13

**Authors:** Ram Pada Das, Minati Nayak, Beena Gobind Singh, Koushik Majee, Amit Kunwar

**Affiliations:** ^1^ Chemical Sciences Discipline, Homi Bhabha National Institute, Anushaktinagar, Mumbai, Maharashtra, 400094, India, hbni.ac.in; ^2^ Radiation and Photochemistry Division, Bhabha Atomic Research Centre, Mumbai, Maharashtra, 400085, India, barc.ernet.in; ^3^ Life Sciences Discipline, Homi Bhabha National Institute, Anushaktinagar, Mumbai, Maharashtra, 400094, India, hbni.ac.in

**Keywords:** cellular uptake, curcumin, drug delivery, endocytosis, gelatin, genipin

## Abstract

Gelatin‐based nanoformulations have received special attention for drug delivery applications because of their regulatory acceptability. A thorough understanding of the factors controlling the interaction of gelatin nanocarriers with cellular systems is crucial for their future biomedical applications. The present study addresses the effect of genipin crosslinking on the ability of gelatin nanoparticles (GNPs) to deliver curcumin, a pharmacologically active ingredient from turmeric into lung cancer (A549) cells. Briefly, the methodology was optimized to prepare GNP (15 mg/mL) crosslinked with 0.25, 0.5, and 1.0 mg/mL of genipin (GN‐GNP1‐3, respectively). The crosslinking of GN‐GNP1‐3 was established through UV–VIS, Fourier transform infrared spectroscopy, and circular dichroism measurements. Dynamic light scattering and transmission electron microscopy showed nearly identical hydrodynamic size (165 ± 15 nm) and shape (spherical) for GN‐GNP1‐3. Subsequently, these nanocarriers were loaded with curcumin and evaluated for drug delivery properties (loading efficiency and release kinetics), cellular uptake, cytotoxicity, and associated mechanisms. These studies together revealed that GN‐GNP1‐3 of increasing degree of crosslinking exhibited higher curcumin loading efficiency, facilitated slow and sustained release of curcumin over a prolonged period (80 h) by a non‐Fickian mechanism, and ultimately increased the cellular uptake and the effectiveness (or cytotoxicity) of entrapped curcumin in A549 cells. The pharmacological abrogation investigations established that curcumin‐loaded GN‐GNP3 was internalized within A549 cells through caveolae‐mediated endocytosis. In conclusion, genipin crosslinking of gelatin‐based nanocarriers seemed to be a novel strategy to increase the cellular uptake cum effectiveness of a hydrophobic payload like curcumin.

## 1. Introduction

In recent years, drug delivery systems have emerged as a novel strategy to enhance the effectiveness of pharmacological agents by facilitating their controlled release at the target site [1, 2]. Among the wide array of delivery systems, the gelatin‐based carriers have attracted a lot of attention for drug delivery applications [3–6]. This is primarily because gelatin is an FDA‐approved excipient and is used in various pharmaceutical/nutraceutical preparations [7–9]. Biochemically, gelatin is a hydrolyzate obtained from the alkaline or acid hydrolysis of collagen protein [7–9]. It possesses several features such as biocompatibility, biodegradability, low immunogenicity, and amicability for surface functionalization, all of which are required to design an ideal drug delivery system [7–9]. Accordingly, researchers have explored gelatin as a drug delivery system in various forms such as nanoparticles, microspheres, gels, and capsules [3–9]. Among these, gelatin nanoparticles (GNPs) are highly promising due to their size‐restricted passive targeting ability. The methods commonly employed for preparing GNPs include desolvation, thermal denaturation, nanoprecipitation, emulsification, and coacervation, among others [7–9]. Moreover, composite nanocarriers including gelatin with other excipients such as Pluronic, lipids, and carboxymethyl cellulose are also reported [10–12]. Such composite nanocarriers of gelatin have mostly shown superior stability or mechanical strength [10–12]. The efficiency of a nanocarrier for drug delivery application depends on its interaction with the cellular system at the target site. Indeed, recent studies suggest that colloidal properties (hydrodynamic size and surface charge) and surface characteristics (hydrophobicity, rigidity/elasticity, and functionalization, among others) of nanoparticles can influence their interactions with cellular systems and thereby alter the delivery parameters (loading, release, and cellular uptake) of the entrapped payload [13, 14]. Therefore, a thorough understanding of the factors controlling the interactions of GNP with cellular systems is crucial for the effective design of gelatin‐based drug formulations for biomedical applications [13, 14].

In this context, our group has earlier reported the utility of GNP for enhancing the cellular uptake, as well as the cytotoxic effect of anticancer agents like doxorubicin, irinotecan, and curcumin against cancer cells [15–17]. These studies have indicated that the size and hydrophobicity of GNP significantly influence the loading, release, and cellular uptake of the payload [15–17]. Furthermore, our group has also reported that albumin nanoparticles having different modes of crosslinking (chemical vs. physical) differ in their ability to internalize within the target cells primarily due to the differences in their rigidity [18]. On similar lines, researchers have shown that the degree and nature of crosslinking can influence the drug delivery properties of GNP [19, 20]. While crosslinking strategies are known to improve the drug delivery properties of nanoparticles, the inherent toxicity of crosslinkers remains a major concern [21]. Recently, genipin has emerged as a biocompatible natural crosslinking agent for protein‐based nanocarriers, including GNP [21–23]. It forms covalent bonds with the primary amine groups present in the lysine residues of proteins, thereby achieving crosslinking [21]. However, to the best of our knowledge, there are no such reports correlating the extent of genipin crosslinking of GNP with the loading and cellular uptake of hydrophobic drugs. Accordingly, the objective of the present study was to understand the effect of genipin crosslinking on the delivery properties including cellular uptake of GNP using curcumin as a model hydrophobic payload. Curcumin, an active ingredient from turmeric, is one of the most extensively researched phytochemicals for various pharmacological properties, including anticancer activity [24, 25]. However, the major challenge in clinical translation of curcumin as a therapeutic agent or drug is its very low bioavailability [26]. The low bioavailability of curcumin has been primarily attributed to its tendency to undergo faster chemical and metabolic degradation and poor cellular uptake [26]. In the present study, GNP prepared using the desolvation technique was crosslinked with varying amounts of genipin. Following this, genipin‐crosslinked GNPs were loaded with curcumin and evaluated for drug delivery properties, including loading efficiency, release kinetics, cellular uptake, cytotoxicity, and associated mechanisms. The findings of the present study are expected to be useful for future design of biopolymeric nanocarriers for hydrophobic drugs, including curcumin, for increasing their bioavailability and therapeutic efficacy.

## 2. Methods

### 2.1. Chemicals

Gelatin (Type A, bloom size = 80–120), curcumin, genipin, sodium chloride, sodium hydrogen phosphate (monobasic and dibasic), potassium hydrogen phosphate, potassium chloride, uranyl acetate, Triton X‐100, 3‐(4,5‐dimethyl‐2‐thiazole)‐2,5‐diphenyltetrazolium bromide (MTT), and dimethyl sulfoxide (DMSO) were procured through local agents from Sigma‐Aldrich (Merck, USA). Dynasore, filipin, and chlorpromazine were obtained from Cayman Chemical (USA). Dulbecco’s modified Eagle medium (DMEM), trypsin–EDTA, penicillin, fetal bovine serum (FBS), and dialysis membrane‐60 of diameter 16 mm and molecular cutoff 12–14 kDa were purchased from Himedia (India). The Bradford protein assay kit was purchased from Bangalore Genei (India). Ethanol and methanol were purchased from Sisco Research Laboratories (India). The solutions were prepared using water from the Millipore water purification system. The freshly prepared solutions were used for all the experiments.

### 2.2. Synthesis of GNP

GNP was synthesized by a one‐step desolvation technique. Briefly, varying amounts (15–50 mg/mL) of gelatin were dissolved in 2 mL of water under mild heating. Following this, acetone (2 mL) or ethanol (2–10 mL) was added dropwise to the gelatin solution under constant stirring at 100 RPM for 2 h. The resulting solution was centrifuged at 12,000 RPM for 10 min. The precipitate/pellet obtained was re‐suspended into water and used as a GNP solution.

### 2.3. Genipin Crosslinking With GNP

The varying amount of genipin (0.5, 1, and 2 mg) dissolved in 200 μL of water:acetone mixture (1.0:0.5) was added to the 1.8 mL of GNP solution (containing 30 mg of gelatin equivalent) as prepared above and incubated at 40°C for 30 min under constant stirring of 100 RPM. Genipin was purposefully dissolved in the water:acetone mixture, considering its limited solubility in the aqueous medium, and thus to avoid its precipitation during the conjugation reaction. The appearance of blue color indicated crosslinking of GNP with genipin. The genipin‐crosslinked GNP was purified by dialyzing the reaction mixture against water using a dialysis tube (MWCO 12–14 kDa). Henceforth, GNP crosslinked or functionalized with 0.25, 0.5, and 1.0 mg/mL of genipin will be referred to as GN‐GNP1, GN‐GNP2, and GN‐GNP3, respectively. A portion of the solution of GN‐GNP3 was lyophilized and subjected to FTIR characterization (IR Affinity 1, Shimadzu, Japan) in the wavelength range of 500–4000 cm^−1^ to validate the crosslinking.

### 2.4. Determination of the Degree of Crosslinking

The degree of crosslinking was expressed as the percentage increase in the optical density of GN‐GNP1‐3 against plain GNP at 600 nm (absorption maximum corresponding to blue‐colored genipin–amino acid conjugate) recorded using a UV–VIS spectrophotometer (V‐630, JASCO, Japan) [23].

### 2.5. Characterization of GNP and GN‐GNP

The hydrodynamic size and surface charge of GNP/GN‐GNP1‐3 were determined using their colloidal solutions through dynamic light scattering (DLS) and zeta sizer (Litesizer DLS 500, Anton Paar, Graz, Austria) as mentioned in the previous reports [15–18]. The morphology and the core size of GNP/GN‐GNP1‐3 were determined by performing transmission electron microscopy (TEM, JEM 1400 PLUS, JEOL, Tokyo, Japan) using a previously reported method [15–18]. The secondary structure of GN/GN‐GNP1‐3 was determined by recording circular dichroism (CD) spectrum (Biologic MOS450‐SFM 300, France) in the wavelength range of 200–270 nm as per the previous method [15–18].

### 2.6. Loading of Curcumin Into GN‐GNP

Briefly, 5 mg/mL of curcumin solution was prepared in ethanol. The desired amount of curcumin was added to 2 mL of GNP/GN‐GNP1‐3 solution (containing 30 mg of gelatin equivalent) and stirred at room temperature for 2 h under constant stirring of 100 RPM. Unbound curcumin was removed by dialyzing the reaction mixture using a dialysis tube (MWCO 12–14 kDa) and water as the dialyzing solvent for 12 h. The resulting solution contained curcumin‐loaded GNP/GN‐GNP1‐3. The amount of curcumin entrapped within GNP/GN‐GNP1‐3 was estimated by UV–Visible spectroscopy (V‐630, JASCO, Japan) according to the method reported earlier [17, 18]. Furthermore, the encapsulation efficiency of curcumin was calculated by the following equation:

(1) Encapsulation efficiency=Amount of CUR entrapped into nanocompositeAmount of CUR added×100.

### 2.7. In Vitro Curcumin Release

The release of curcumin from GNP/GN‐GNP1‐3 was studied under reservoir‐sink conditions [15–18]. Briefly, 2 mL of the curcumin‐loaded GNP/GN‐GNP1‐3 in a dialysis tube (MWCO 12–14 kDa) was placed in 100 mL of release medium (phosphate‐buffered saline at pH 7.4 containing 0.1% Triton X‐100) under continuous stirring (100 RPM) at 37°C to mimic the reservoir and sink, respectively. The pH of the sink was purposefully kept at 7.4 (close to physiological pH) in order to avoid any interference from pH (stimuli) responsive release of drug due to the degradation of gelatin nanocarriers [15–18]. Furthermore, 0.1% Triton X‐100 was added to the sink to solubilize the hydrophobic curcumin, which is released from the nanoparticles. 1 mL of the sink medium was withdrawn at regular time intervals, and the amount of curcumin was determined by monitoring absorbance at 420 nm using a multiwell microplate reader (Synergy Hybrid H1, BioTek, Vermont, USA) against the standard plot prepared under similar conditions.

### 2.8. Cell Culture

The human lung adenocarcinoma (A549) cell line was chosen as the cellular model, as curcumin is well tested for anticancer effects in this cell type [27]. The cells obtained from the National Centre for Cell Science (Pune, India) were cultured in DMEM medium containing 10% FBS, 100 U/mL penicillin, and 100 μg/mL streptomycin and maintained at 37°C in a humidified incubator (MCO‐230AICUVH, Panasonic, Japan) with 5% CO_2_.

### 2.9. Determination of Curcumin Uptake in Cells Through GNP/GN‐GNP

The cellular uptake of curcumin through DMSO/GNP/GN‐GNP1‐3 was studied by following the previously reported methodology from our group [17, 18]. Briefly, the methodology involved treatment of A549 cells (∼1 × 10^6^ cells/mL) with 25 μM of curcumin equivalent through DMSO/GNP/GN‐GNP1‐3 for 6 h, followed by the extraction of intracellular curcumin into methanol, recording the absorbance of methanolic curcumin at 420 nm, and estimation of curcumin against the standard curve under similar conditions. The cellular uptake was normalized with respect to total cellular protein and expressed as the amount of curcumin per mg of protein.

### 2.10. Fluorescence Microscopy

The intracellular delivery of curcumin through DMSO/GN‐GNP3 was also monitored through fluorescence imaging of the cells using a fluorescence microscope (IX83P2ZF, Olympus, Tokyo, Japan) coupled with a camera and band pass filters. For this, cells (1 × 10^4^) were grown on coverslips, treated with 25 μM of curcumin equivalent through DMSO/GN‐GNP3 for 6 h, and processed as described in previous reports [17, 18]. In order to establish the mechanism of cellular uptake, the cells were preincubated (2 h) with the inhibitors (Dynasore—20 μM, Filipin—5 μM, Chlorpromazine—10 μM) specific for various endocytosis pathways and then treated with curcumin (25 μM)‐loaded GN‐GNP3 followed by fluorescence imaging using a FITC filter. The images were analyzed for mean fluorescence intensity (MFI) using ImageJ software (ImageJ 1.54 g).

### 2.11. Cytotoxicity Assay

The cytotoxicity of curcumin (25 μM) through various vehicles (DMSO/GNP/GN‐GNP1‐3) was evaluated by MTT assay [15–18].

### 2.12. Statistical Analysis

Each experiment was performed in triplicate and repeated at least two times. The results are presented as mean ± SD (*n* = 3) of an independent measurement. The statistical significance of the means of two groups was evaluated by Student’s *t*‐test (Origin, Version 6.1, one‐tailed, two‐sample unequal variance), and *p* values < 0.05 were considered significant.

## 3. Results

### 3.1. Synthesis and Characterization of GN‐GNP

GNP was prepared by employing a well‐established nanoprecipitation/desolvation technique [16]. Initially, in order to optimize the choice of antisolvent medium, the aqueous solution containing a fixed concentration (15 mg/mL) of gelatin was subjected to nanoprecipitation by mixing it with increasing volumetric ratios of ethanol or acetone as the antisolvent medium. The formation of nanoparticles under each of the above preparation methods was monitored visibly by observing the turbidity of the reaction mixture. Notably, the addition of ethanol to the aqueous solution of gelatin in the volumetric ratios of 1:1 and 2.5:1 did not show any turbidity (Table S1). Furthermore, on increasing the volumetric ratio of ethanol versus aqueous gelatin to 5:1, the reaction mixture showed turbidity, suggesting the formation of colloidal particles (Table S1). On the other hand, a similar process using a 1:1 volumetric ratio of acetone and aqueous gelatin showed visible turbidity, suggesting the formation of GNP. Accordingly, during all the subsequent studies, acetone was employed as the antisolvent medium for the preparation of GNP. Next, the concentration of gelatin required for forming GNP of sufficient yield and desired size was optimized. Briefly, the aqueous solution containing increasing concentrations (7.5–50.0 mg/mL) of gelatin was desolvated with acetone (1:1, v/v) and subjected to DLS characterization for the confirmation of GNP formation. Notably, the lower concentration (7.5 mg/mL) of gelatin did not yield a detectable number of nanoparticles (Table S2). However, gelatin in the concentration range of 15–50 mg/mL yielded stable GNP whose hydrodynamic size increased with increasing concentration of gelatin (Table S2). Alternatively, the zeta potential of GNP did not show any significant change irrespective of the concentration of gelatin (Table S2). The hydrodynamic size and zeta potential of GNP prepared from 15 mg/mL of gelatin were ∼150 nm and ∼−9 mV, respectively. Since biopolymeric nanoparticles in the size range of 150 nm are considered most suitable for drug delivery properties, gelatin at a concentration of 15 mg/mL was fixed for the subsequent surface functionalization or crosslinking with genipin [15–18]. Briefly, the freshly prepared GNPs were incubated with the three increasing concentrations (0.25, 0.5, and 1.0 mg/mL) of genipin to achieve the increasing degree of crosslinking (GN‐GNP1‐3). The concentration of genipin up to 1 mg/mL was purposefully chosen as genipin in this concentration range is completely nontoxic [21, 28, 29]. The resulting mixture after purification as described in the method section was examined for crosslinking by visually monitoring the color change, as well as by recording the UV–VIS absorption and FTIR spectrum. Notably, the purified reaction mixture exhibited the characteristic blue coloration with the corresponding absorption peak at 600 nm, confirming the crosslinking of genipin with GNP (GN‐GNP1‐3) (Figure 1(a)). It can also be seen from the figure that the optical density of GN‐GNP1‐3 at 600 nm increased as a function of genipin concentration, suggesting an increasing degree of crosslinking or conjugation (Figure 1(a)). Furthermore, the ninhydrin assay is the most commonly used assay for the determination of crosslinking by any agent involving amine groups [30]. Accordingly, attempts were also made to determine the percentage of amine content involved in crosslinking by employing the ninhydrin assay [30, 31]. It is worth mentioning that ninhydrin reaction led to the aggregation of plain GNP, as well as of GN‐GNP1‐3, and thus renders them unsuitable for absorption measurement due to very high light scattering (Figure S1). This observation is explainable considering that the ninhydrin assay requires the sample to be incubated with ninhydrin in the water:organic solvent mixture (50/50, v/v) at 90°C [31]. The high temperature along with the presence of organic solvent is well known to induce aggregation of proteinaceous material. Accordingly, the ninhydrin assay was not suitable to determine the degree of crosslinking of GN‐GNP1‐3 nanoparticles. The plot of the percentage increase in the absorbance of GN‐GNP1‐3 at 600 nm as a function of genipin concentration indicated that the absorbance at 600 nm increased linearly with increasing concentration of genipin and almost saturated at the highest tested concentration of 1 mg/mL of genipin and thus justifying the attainment of the maximum degree of crosslinking (Figure 1(b)). Further to validate the above observation, the FTIR spectrum of the lyophilized sample of GN‐GNP3 was compared with those of GNP, neat gelatin, and neat genipin (Figure 1(c)). As expected, the neat gelatin showed the characteristic amide A (N‐H stretching), amide B (N‐H stretching), amide I (C=O and C‐N stretching), amide II (N‐H deformation and C‐H stretching), and amide III (C‐N stretching and N‐H bending) bands at 3430, 2930, 1630, 1530, and 1230 cm^−1^ wave numbers, respectively [32]. The FTIR spectrum of GNP also showed all the important features of gelatin. The FTIR spectrum of neat genipin showed prominent absorption peaks corresponding to C=O stretching of the carboxymethyl group at 1685 cm^−1^, C=C stretching of the olefin ring at 1618 cm^−1^, and C‐H stretching bands in the region around 2873–2952 cm^−1^.

Figure 1(a) Representative UV–VIS spectra of GNP, GN‐GNP1, GN‐GNP2, and GN‐GNP3 in water. (b) The plot shows the percentage increase in absorbance @600 nm of the purified GN‐GNP solution as a function of the concentration of genipin used in the crosslinking reaction. The percent absorbance change of GN‐GNP was calculated with respect to the absorbance @600 nm of GNP. The results are presented as the average ± SD (*n* = 3). (c) The plot shows the FTIR spectra of neat gelatin, neat genipin, GNP, and GN‐GNP3. ^∗^Important IR bands of individual samples as mentioned in the text. (d) The decay correlation function of GNP, GN‐GNP1, GN‐GNP2, and GN‐GNP3 in water as obtained from DLS studies.

**Figure:**
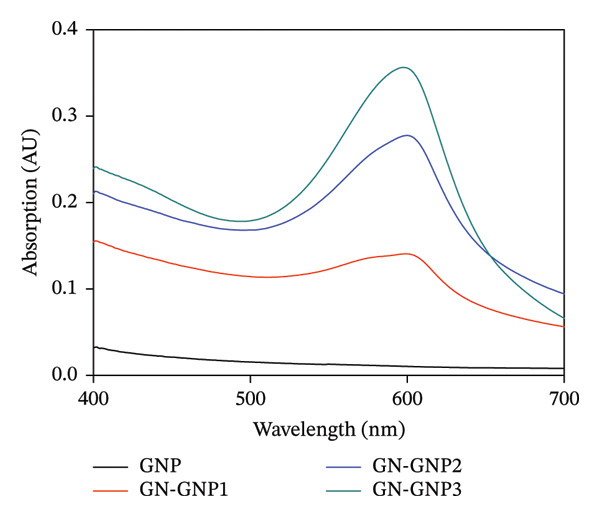
(a)

**Figure:**
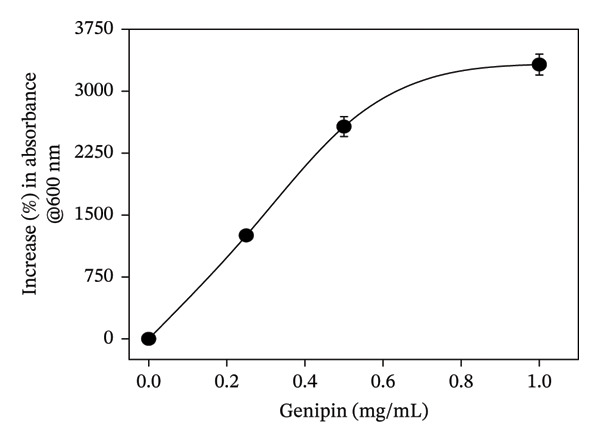
(b)

**Figure:**
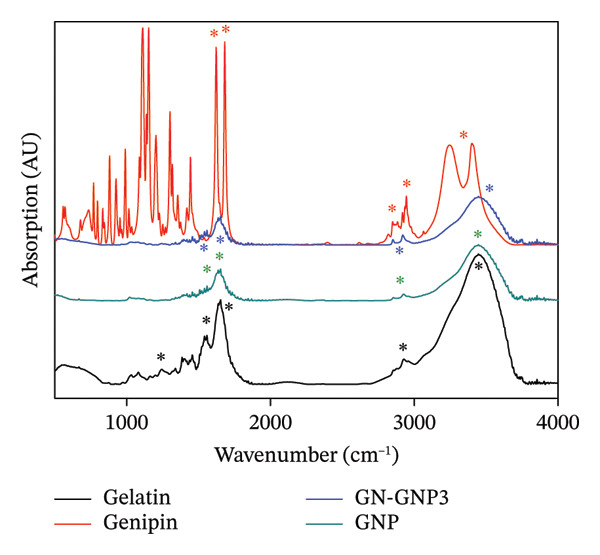
(c)

**Figure:**
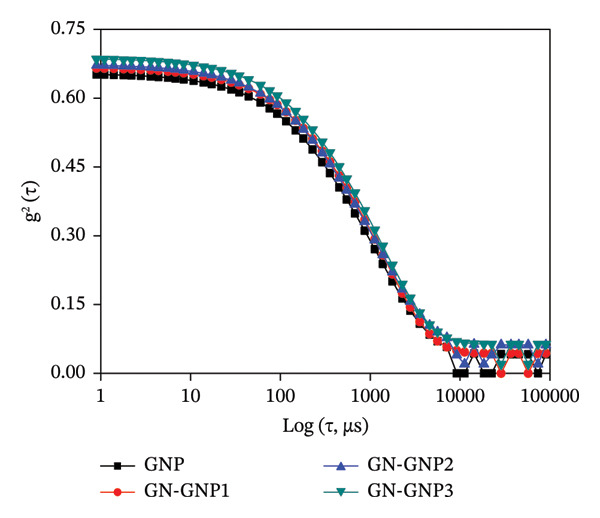
(d)

Additionally, a double absorption peak between 3246 and 3405 cm^−1^ in the FTIR spectrum of genipin could be assigned to the overlapping aromatic C–H and O–H vibration bands. Notably, GN‐GNP3 showed all the characteristic peaks of GNP; however, the peaks at 2850 and 2930 cm^−1^ appeared reinforced as compared to GNP, which is attributed to the contribution from C‐H stretching vibration of genipin. Additionally, the FTIR spectrum of GN‐GNP3 showed the disappearance of the characteristic double absorption peaks (overlapping aromatic C–H and O–H vibration bands) of genipin in the 3246–3405 cm^−1^ region. Together, the above results confirmed the conjugation of genipin with GNP. Furthermore, to understand the effect of the varying degree of crosslinking on the colloidal properties, the freshly prepared GN‐GNP1, GN‐GNP2, and GN‐GNP3 were characterized for hydrodynamic size and surface charge (Table 1, Figure 1(d) and S2). The results indicated a marginal increase in the hydrodynamic size of GNP following crosslinking with genipin in a concentration‐dependent manner. For instance, the hydrodynamic size of GN‐GNP3 representing the highest degree of crosslinking was ∼185 nm. The zeta potential of GN‐GNP did not show any significant variation with the increasing degree of crosslinking and was similar (∼−9 mV) to that of GNP (without genipin crosslinking).

**Table 1:** Effect of the increasing degree of crosslinking with genipin on the colloidal and curcumin release parameters of GNP (15 mg/mL).

Sample ID	Genipin concentration (mg/mL)	Hydrodynamic size (nm)	Scattering counts (kcps)	PI	Zeta potential (mV)	*k* (h^−1^) value from Peppas equation	*n* value from Peppas equation
GNP	0	150 ± 8	24.3	0.13	−9.45	11.7	0.37
GN‐GNP1	0.25	161 ± 11	27.8	0.14	−8.78	10.8	0.42
GN‐GNP2	0.50	170 ± 10	28.3	0.15	−8.12	6.6	0.57
GN‐GNP3	1.00	185 ± 17	39.6	0.12	−8.89	7.3	0.56

Abbreviation: PI, polydispersity index.

### 3.2. Surface Morphology and Secondary Structures of GN‐GNP

**Figure 2:**
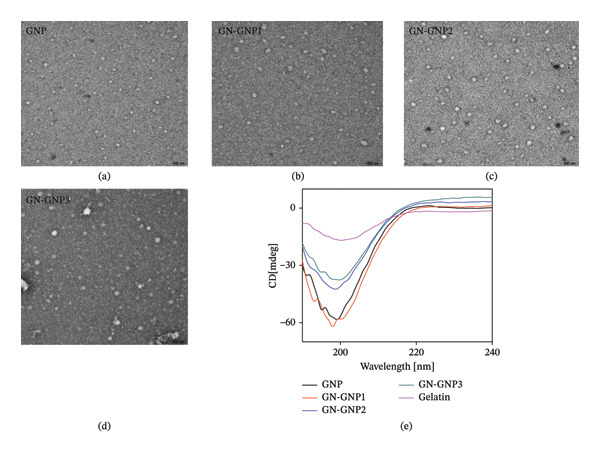
Representative TEM images of GNP (a), GN‐GNP1 (b), GN‐GNP2 (c), and GN‐GNP3 (d). The plot (e) shows the representative CD spectra of gelatin, GNP, GN‐GNP1, GN‐GNP2, and GN‐GNP3 in water.

In order to know the morphology, the freshly prepared GNP, GN‐GNP1, GN‐GNP2, and GN‐GNP3 were characterized through TEM (Figures 2(a), 2(b), 2(c), 2(d)). This analysis revealed that GNP, GN‐GNP1, GN‐GNP2, and GN‐GNP3 maintained a spherical morphology with the core size of 81 ± 17, 91 ± 31, 120 ± 18, and 133 ± 33 nm, respectively. The marginal increase in the core size of GN‐GNP as a function of the crosslinking agent is in agreement with DLS observation as discussed in the previous section. Further to understand the impact of varying degrees of crosslinking on the secondary structure, GN‐GNP was subjected to CD analysis. The results presented in Figure 2(e) indicated that the CD spectra of neat gelatin exhibited the characteristic ellipticity band of a triple helix and a random coil at ∼222 and ∼200 nm, respectively [22]. The CD spectra of GNP showed significant alterations as compared to those of neat gelatin, with a marked increase in the ellipticity at ∼200 nm. This clearly suggested the increase of the random coil structure during the conversion of gelatin into GNP. Interestingly, genipin crosslinking of GNP showed a concentration‐dependent decrease in the ellipticity corresponding to a random coil structure.

Thus, it can be inferred that genipin crosslinking was primarily restricted to the random coil region of GNP probably due to easily accessible lysine residues.

### 3.3. Curcumin Loading Into GN‐GNP

After optimizing the preparation of GN‐GNP of varying degrees of crosslinking, the method was extended for the loading of curcumin within GNP, GN‐GNP1, GN‐GNP2, and GN‐GNP3. Briefly, curcumin was encapsulated within GN‐GNP through the adsorption method by incubating GNP/GN‐GNP (containing 30 mg of gelatin equivalent) with curcumin as described in the method section. In order to estimate the maximum loading efficiency, the encapsulation was performed at varying curcumin concentrations for each of GNP, GN‐GNP1, GN‐GNP2, and GN‐GNP3. Following this, the amount of curcumin encapsulated within GNP, GN‐GNP1, GN‐GNP2, and GN‐GNP3 was estimated as mentioned in the method section. The plot of the amount of curcumin encapsulated versus the amount of curcumin incubated (preloading) with the nanoparticles indicated that irrespective of the degree of crosslinking, the loading of curcumin for a fixed mass of nanoparticles (loading efficiency) increased with the increasing concentration of curcumin used in the loading reaction and almost saturated at a curcumin concentration of 3 mg/mL in the loading reaction (Figure 3(a)). Alternatively, encapsulation efficiency (amount of curcumin entrapped per unit mass of curcumin used in the loading reaction) decreased with the increasing concentration of curcumin in the loading reaction and reached the final value of 31.6 ± 1.6%, 36.0 ± 1.5%, 41.3 ± 1.9%, and 48.3 ± 2.5%, respectively, for GNP, GN‐GNP1, GN‐GNP2, and GN‐GNP3 at a curcumin concentration of 3 mg/mL in the loading reaction (Figure 3(b)).

Figure 3(a) The plots show the variation of the amount of curcumin loaded into various nanoparticles (GNP, GN‐GNP1, GN‐GNP2, and GN‐GNP3) as a function of the concentration of curcumin present in the loading reaction. The nanoparticles (GNP, GN‐GNP1, GN‐GNP2, and GN‐GNP3) contained 30 mg of gelatin equivalent. (b) The plots show the variation of encapsulation efficiency of various nanoparticles (GNP, GN‐GNP1, GN‐GNP2, and GN‐GNP3) as a function of the concentration of curcumin present in the loading reaction. (c) In vitro release kinetics of curcumin from GNP, GN‐GNP1, GN‐GNP2, and GN‐GNP3 under reservoir‐sink conditions. The sink condition represented water containing 0.1% Tx‐100. The results are presented as mean ± SD (*n* = 3).

**Figure:**
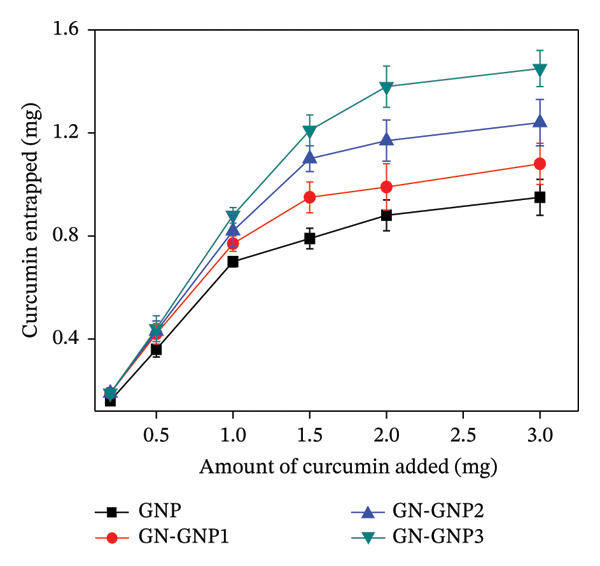
(a)

**Figure:**
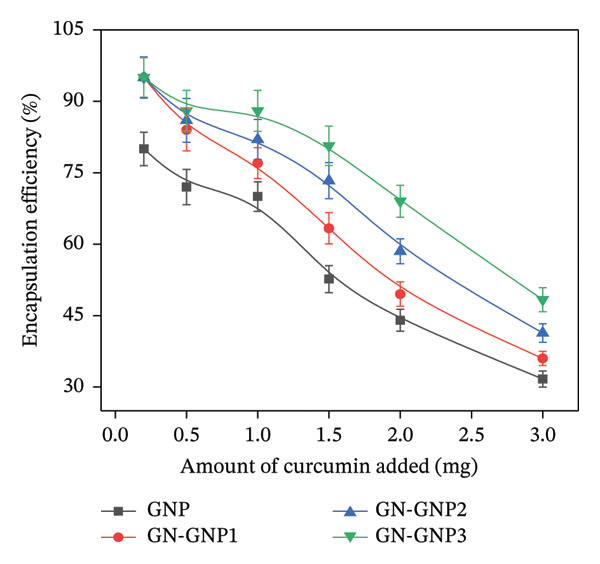
(b)

**Figure:**
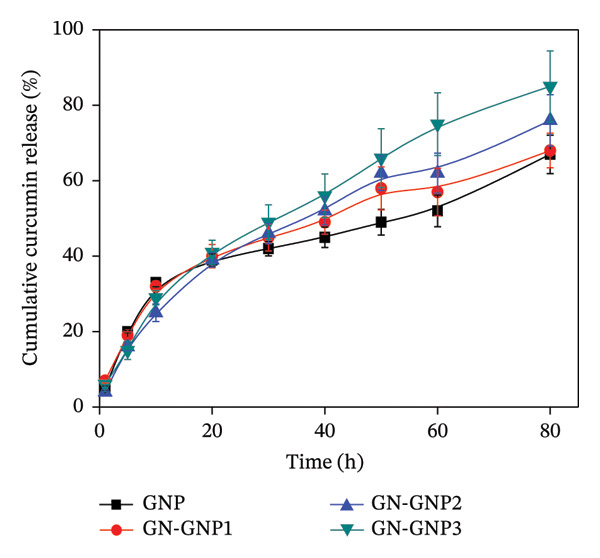
(c)

The above results are explainable considering that the amount of nanocarrier may be sufficient to encapsulate curcumin at lower loading concentrations. However, with increasing curcumin concentration in the loading reaction, although the amount of curcumin entrapped per unit mass of carrier may increase, the amount of nanocarrier may become a limiting factor for the amount of curcumin available for encapsulation, thus leading to a decrease in the encapsulation efficiency. Furthermore, it can also be seen from the figure that the maximum loading efficiency of GN‐GNP with varying degrees of crosslinking followed the order of GNP < GN‐GNP1 < GN‐GNP2 < GN‐GNP3. This clearly suggested that genipin crosslinking increased the loading efficiency of GNP for curcumin in a concentration‐dependent manner with GN‐GNP3 showing the highest loading efficiency of 4.83 ± 0.33%.

### 3.4. In Vitro Release Kinetics of Curcumin From GN‐GNP

Next, the release of curcumin from GN‐GNP of varying degrees of crosslinking was studied using the reservoir‐sink model as explained in the method section. According to Figure 3(c), the release of curcumin from GN‐GNP followed a biphasic trend irrespective of the degree of crosslinking with all four nanocarriers, viz., GNP, GN‐GNP1, GN‐GNP2, and GN‐GNP3, showing the initial faster release up to 20 h followed by slower release during the extended period up to 80 h. For instance, all the four nanocarriers, viz., GNP, GN‐GNP1, GN‐GNP2, and GN‐GNP3 exhibited ∼40% of curcumin release by 20 h and of 67 ± 5%, 68 ± 4.6%, 76 ± 6.8%, and 85 ± 9.4% curcumin release by the end of 80 h. This clearly suggested that the degree of crosslinking of GN‐GNP affected the release of curcumin during the later (slower) phase of release kinetics. It is also worth mentioning that the release of curcumin from the above carriers irrespective of the degree of crosslinking was gradual and did not show saturation (or plateau).

Furthermore, polymeric nanocarriers are generally known to show complex drug release kinetics, which may involve both Fickian and non‐Fickian mechanisms [17]. Accordingly, the Korsmeyer–Peppas model (Equation (2)), which takes into account both Fickian and non‐Fickian release mechanisms, is commonly employed to understand the drug release mechanisms of polymeric nanocarriers [22]:

(2) CtC∞=ktn.

Here, *C*_*t*_/*C*_*∞*_ represents the fraction of curcumin released at a given time (*t*). The other parameters like *k* and *n* represent the release rate and release exponent, respectively. As per this model, the value of *n* indicates the release mechanism. For instance, *n* ≤ 0.45 suggests the release of entrapped drug through a Fickian mechanism generally due to the diffusion of the release medium into the nanocarrier [17], whereas *n* > 0.45 is indicative of a non‐Fickian mechanism involving both diffusion of the release medium into the nanocarriers, as well as the relaxation of the nanocarrier releasing the entrapped drug [17]. Notably, nonlinear fitting of the release kinetics data corresponding to the first 60% of curcumin release from GNP, GN‐GNP1, GN‐GNP2, and GN‐GNP3 to Korsmeyer–Peppas equation indicated the respective *n* values of 0.37, 0.42, 0.57, and 0.56 (Figures 4(a), 4(b), 4(c), 4(d)). This clearly suggested that the release mechanism of curcumin from GN‐GNP shifted from Fickian to non‐Fickian with the increasing degree of crosslinking. Moreover, the four nanocarriers, viz., GNP, GN‐GNP1, GN‐GNP2, and GN‐GNP3, showed *k* values of 11.7, 10.8, 6.6, and 7.3 h^−1^, respectively, suggesting that the average rate of release of curcumin from GN‐GNP decreased with increasing degree of crosslinking. As the gradual and slow release of a drug over an extended period is indicative of sustained release behavior, it is inferred that genipin crosslinking improved the ability of GNP for maintaining the sustained release of curcumin over a long duration, an essential requirement for a drug delivery system or nanocarriers to be effective under clinical settings [7–9, 15, 16]. The overall slower rate of release of curcumin from GN‐GNP2 and GN‐GNP3 over an extended period (80 h) is attributed to the stronger binding of curcumin due to the availability of a higher number of hydrophobic pockets.

Figure 4Plots show the mathematical fitting of the 60% release kinetics of curcumin from (a) GNP, (b) GN‐GNP1, (c) GN‐GNP2, and (d) GN‐GNP3 according to the Korsmeyer–Peppas model (Equation (2)).

**Figure:**
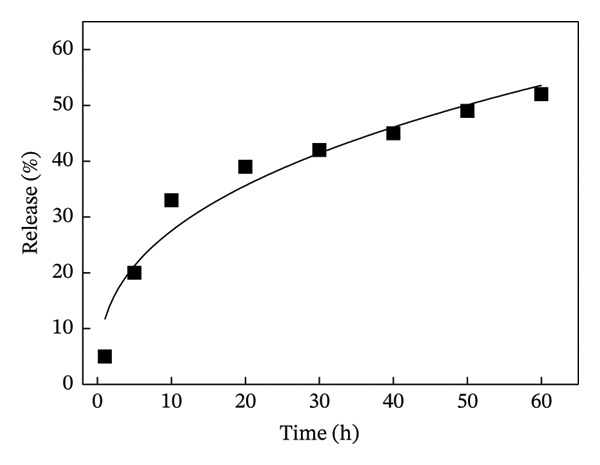
(a)

**Figure:**
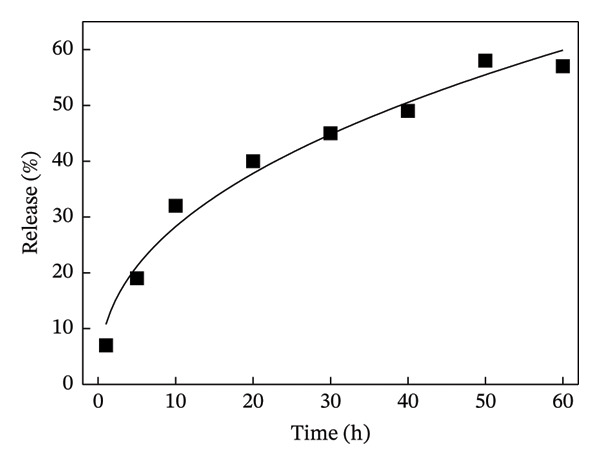
(b)

**Figure:**
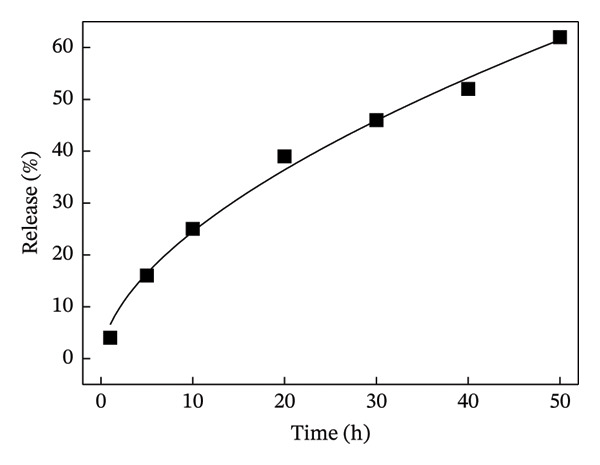
(c)

**Figure:**
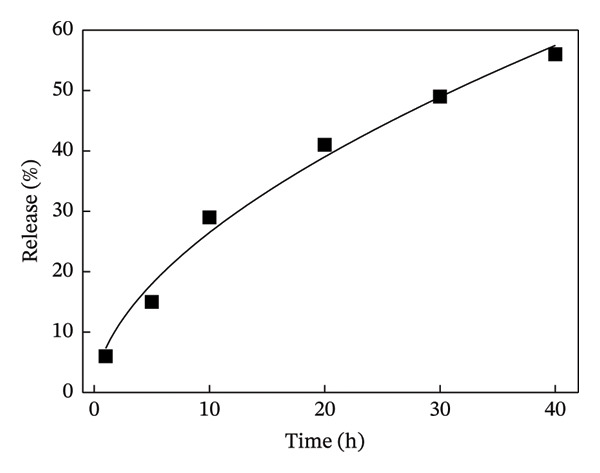
(d)

### 3.5. Cellular Uptake of Curcumin‐Loaded GN‐GNP and the Associated Mechanism

**Figure 5:**
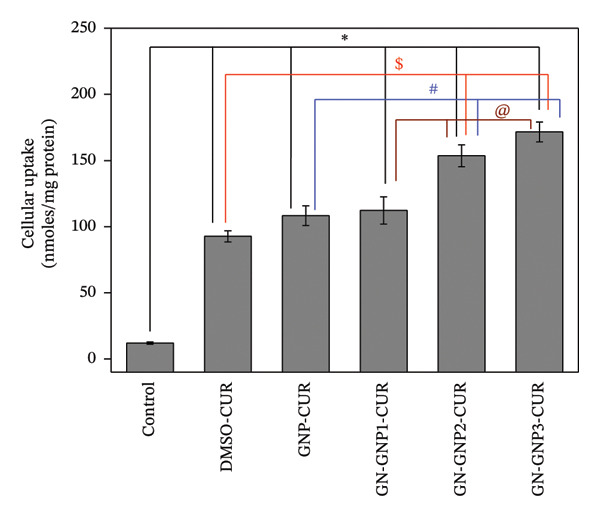
The plot shows the intracellular level of curcumin in cells following treatment with 25 μm curcumin equivalent through different vehicles (DMSO, GNP, GN‐GNP1, GN‐GNP2, and GN‐GNP3). The results are presented as mean ± SD (*n* = 3). ^∗^ *p* < 0.05 as compared to control; ^$^ *p* < 0.05 as compared to DMSO‐CUR; ^#^ *p* < 0.05 as compared to GNP‐CUR and ^@^ *p* < 0.05 as compared to GN‐GNP1‐CUR. CUR—curcumin. The final concentration of DMSO in the cell culture medium was 0.15%.

The uptake of the drug‐loaded nanocarriers into target cells is also an important criterion to judge their utility as a drug delivery system [13–18]. Accordingly, the cellular uptake of curcumin‐loaded GNP, GN‐GNP1, GN‐GNP2, and GN‐GNP3 was investigated in the human lung cancer (A549) cells using a previously optimized methodology from our group [17, 18]. For these studies, cells were treated at a fixed curcumin equivalent concentration of 25 μM through various GNP for 6 h. For comparison, curcumin (25 μM) through DMSO (0.15%) was also included in the study. The quantification of intracellular curcumin indicated that cellular uptake of curcumin through various carriers followed the order of DMSO∼GNP∼GN‐GNP1 < GN‐GNP2 < GN‐GNP3. This confirmed the ability of GN‐GNP to increase the intracellular uptake of curcumin with an increasing degree of crosslinking (Figure 5). Notably, GN‐GNP3 with the highest degree of crosslinking exhibited almost ∼1.58‐fold (*p* < 0.05) and ∼1.85‐fold (*p* < 0.05) higher delivery of curcumin as compared to GNP (without crosslinking) and DMSO (organic solvent).

**Figure 6:**
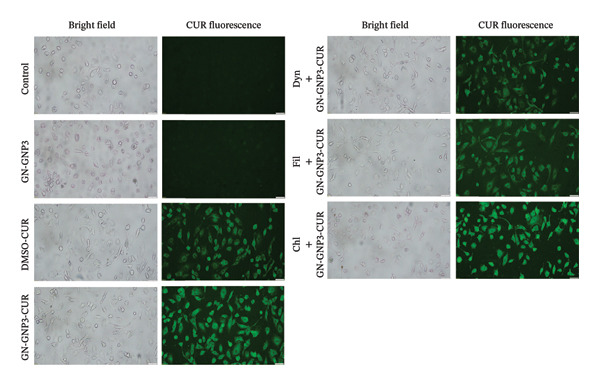
The effect of various inhibitors (Dyn—dynasore; Fil—filipin; Chl—chlorpromazine) of the endocytosis pathway on the cellular delivery of curcumin (25 μm) in A549 cells through GN‐GNP3. The fluorescence images were captured after excitation using a FITC filter equipped with a microscope (Olympus IX83). Magnification 20X. CUR—curcumin.

Further to validate the above observations, cells treated with DMSO‐CUR and GN‐GNP3‐CUR were imaged using a fluorescence microscope and quantified for MFI (*n* = 25 cells per field) (Figure 6 and S3). The representative images and MFI of cells treated with DMSO‐CUR and GN‐GNP3‐CUR showed significantly higher fluorescence emission as compared to the control and blank GN‐GNP3 groups. As expected, the group treated with GN‐GNP3‐CUR showed significantly higher fluorescence than the DMSO‐CUR group, thus confirming the ability of GN‐GNP3 to increase the intracellular uptake of curcumin. Earlier studies have indicated that soft nanoformulations are internalized within the cells through endocytosis pathways [33–35]. The endocytosis of solid particles is primarily mediated through clathrin and caveolin‐mediated pathways [33–36]. Dynasore, filipin, and chlorpromazine are the well‐known inhibitors of endocytosis pathways [37]. Dynasore is a potent inhibitor of dynamin, a GTPase protein that plays an important role during both clathrin‐ and caveolae‐mediated endocytosis [37, 38]. Filipin mainly blocks caveolae‐mediated endocytosis by disrupting caveolae [37], whereas chlorpromazine mainly blocks clathrin‐mediated endocytosis by disrupting clathrin‐coated pits on the plasma membrane [37].

Therefore, to understand the mechanism of cellular uptake, the effect of the pretreatment (2 h) of the above inhibitors on the cellular uptake of GN‐GNP3‐CUR was studied by fluorescence imaging (Figure 6 and S3). Remarkably, the cells treated with GN‐GNP3‐CUR in the presence of dynasore or filipin showed significantly lower fluorescence than the GN‐GNP3‐CUR group. On the contrary, the fluorescence of cells treated with GN‐GNP3‐CUR in the presence of chlorpromazine did not show any significant difference as compared to the GN‐GNP3‐CUR group. As expected, vehicle control groups including DMSO (0.15%), inhibitor controls, and different GNP without curcumin did not show any significant fluorescence emission (data not shown). The above results thus confirmed that the internalization of GN‐GNP3‐CUR within A549 was mediated through caveolae‐mediated endocytosis.

### 3.6. Cytotoxicity of Curcumin‐Loaded GN‐GNP

**Figure 7:**
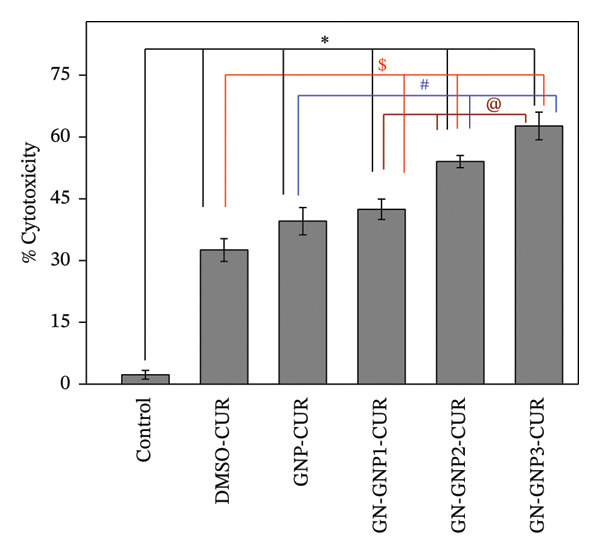
The plot shows the % cytotoxicity in A549 cells following treatment with 25 μM curcumin equivalent of various formulations for 48 h by MTT assay. The results are presented as mean ± SD (*n* = 3). ^∗^ *p* < 0.05 as compared to control; ^$^ *p* < 0.05 as compared to DMSO‐CUR; ^#^ *p* < 0.05 as compared to GNP‐CUR; and ^@^ *p* < 0.05 as compared to GN‐GNP1‐CUR. CUR—curcumin.

Finally, the intended applications of curcumin‐loaded GNP, GN‐GNP1, GN‐GNP2, and GN‐GNP3 may involve their utilization as nanoformulations for improving the efficacy (cytotoxic effect) of curcumin against cancerous cell types [1, 2]. Accordingly, the cytotoxic effect of the above formulations at an identical curcumin concentration (25 μM) was investigated in A549 cells after 48 h of treatment by MTT assay (Figure 7). As expected, curcumin delivered through DMSO (0.15%) showed significantly (*p* < 0.05) higher cytotoxicity as compared to control untreated cells. Furthermore, curcumin delivered through GNP led to a marginal increase in the cytotoxic effect as compared to DMSO curcumin, whereas curcumin delivered through GN‐GNP exhibited significantly (*p* < 0.05) higher cytotoxicity than the DMSO group. Furthermore, the cytotoxicity effect of curcumin‐loaded GN‐GNP increased with increasing degree of crosslinking. Notably, curcumin delivered through GN‐GNP3 exhibited almost ∼2‐fold (*p* < 0.05) and ∼3‐fold (*p* < 0.05) higher toxicity than those through GNP (without crosslinking) and DMSO (organic solvent), respectively. The vehicle control groups (0.15% DMSO and equivalent volumes of different nanocarriers without curcumin) did not show any significant cytotoxicity as compared to the control, suggesting their biocompatibility (Figure S4).

## 4. Discussion

**Scheme 1:**
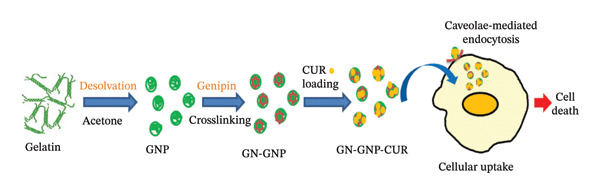
Schematic summarizes the preparation of curcumin‐loaded GNP and its cellular uptake through the endocytosis mechanism.

Drug delivery systems that can enhance the efficacy of the payload by increasing its cellular uptake are highly sought after for the development of pharmaceutical formulations [1, 2, 39]. Despite a significant amount of research carried out in this field, only a few delivery systems based on either liposome or biopolymers (such as albumin) have reached the clinical trial stage primarily because of their biocompatibility [1, 2, 39]. Similarly, gelatin being a biocompatible and FDA‐approved excipient also gains a lot of significance as it will be easier to translate gelatin‐based delivery systems under a clinical setting [7–9]. However, before reaching that stage, the factors influencing drug delivery properties of gelatin‐based carriers need to be thoroughly investigated employing in vitro and in vivo model systems [9, 13, 14]. In this context, apart from the physicochemical properties (such as size, surface charge, and hydrophobicity), the mechanical properties, especially rigidity/elasticity of nanocarriers, have also been suggested to influence their interaction with the cellular system [13, 14, 33–35]. Among various approaches, crosslinking is a widely used strategy for tuning the mechanical properties of nanocarriers [19, 40]. Accordingly, there is a pressing need to understand the influence of crosslinking on the in vitro drug delivery properties (loading, release, and cellular uptake) of GNP for the future design of gelatin‐based effective drug delivery systems. In order to address this, genipin and curcumin were chosen as the crosslinking agent and the model hydrophobic drug, respectively [21, 24, 25]. Initial optimization studies indicated that acetone worked as a better antisolvent than ethanol to achieve control over the hydrodynamic size and polydispersity of GNP following desolvation. It could be due to the lower dielectric constant of acetone than ethanol, favoring efficient removal of water molecules from the surface of gelatin, ultimately leading to self‐assembly or nucleation of gelatin monomers through hydrophobic interactions and the formation of compact and stable nanoparticles as the precipitates [41, 42]. By optimizing the concentrations of gelatin and genipin, GNPs with an increasing degree of crosslinking, viz., GN‐GNP1, GN‐GNP2, and GN‐GNP3, of spherical morphology with hydrodynamic size in the range of 150–180 nm and surface charge of −9 mV were prepared. The conjugation or crosslinking of genipin was established through UV–VIS, FTIR, and CD measurements. The loading of curcumin and the subsequent in vitro studies of drug delivery properties (loading/encapsulation efficiency and release kinetics) indicated that genipin crosslinking significantly altered these parameters associated with GNP. Previously, it has been shown that nanocarriers having a greater number of hydrophobic sites show higher curcumin loading [17, 18]. With a similar analogy, the increase in the loading efficiency of GNP upon genipin crosslinking is attributed to the increase in the number of hydrophobic sites with increasing degree of crosslinking. Furthermore, the biphasic (faster followed by slower) release kinetics of curcumin from GN‐GNP irrespective of the degree of crosslinking suggest that curcumin encapsulated near the surface of GNP probably contributed to the faster component as they would be easily accessible for release through the weakening of hydrophobic forces [43, 44], whereas the curcumin entrapped into the core of GNP is believed to be contributing to the slower component of the release kinetic as they would need the erosion or disruption of the nanoparticles to occur in order to facilitate the release, which is expected to occur slowly over the extended period of time [43, 44]. Additionally, GN‐GNP depending on the degree of crosslinking showed sustained release of curcumin over a long duration of 80 h, which is attributed to their higher loading efficiency. Most remarkably, curcumin‐loaded GN‐GNP depending on the degree of crosslinking exhibited significantly higher cytotoxicity against A549 (lung cancer) cells, with GN‐GNP3 exhibiting ∼2‐fold higher cytotoxicity than GNP (without crosslinking) at an identical curcumin concentration. The enhanced cytotoxicity could be due to the higher uptake of curcumin through the nanoparticles [17, 18]. In order to address this, the cellular uptake of curcumin through various GNP formulations was estimated. As per the results, GN‐GNP depending on the degree of crosslinking significantly increased the cellular uptake of curcumin. However, the relative increase in the uptake of curcumin through GN‐GNP (with increasing degree of crosslinking) was slightly lower than the relative increase in the cytotoxicity of curcumin achieved through these carriers as compared to GNP. This suggested that the enhancement in the cytotoxicity of curcumin through GN‐GNP was probably due to the improved uptake, as well as the ability of GN‐GNP to maintain the sustained release of curcumin over a period of time. Recent studies have established that the cellular uptake of protein nanoparticles is influenced by their hydrodynamic size [15–18]. Therefore, it can be argued that the variation in cellular uptake of GNP, GN‐GNP1, GN‐GNP2, and GN‐GNP3 could be due to difference in their hydrodynamic size. However, it is worth mentioning that all the above four formulations possessed almost similar hydrodynamic size (165 ± 15 nm) but differed with respect to the degree of crosslinking. Thus, it suggests that the difference in the uptake of GNP, GN‐GNP1, GN‐GNP2, and GN‐GNP3 was probably due to the difference in their rigidity acquired during crosslinking. The above assumption is supported by recent studies, which indicate that biopolymeric nanoparticles with increasing rigidity are preferably internalized into cells by favoring the endocytosis process [33–35]. The fluorescence imaging studies in the presence of the inhibitors of endocytosis pathways indeed confirmed that the uptake of GN‐GNP3 within A549 cells was governed through caveolae‐dependent endocytosis. Taken together, GN‐GNP3 with the highest degree of crosslinking appears to have ideal hydrodynamic size and rigidity to improve the cellular uptake in turn cytotoxicity of hydrophobic payload like curcumin as shown in Scheme 1.

## 5. Conclusions

In conclusion, the present study for the first time demonstrates the effect of genipin crosslinking on the in vitro drug delivery properties (loading, release, cellular uptake, and cytotoxicity) of curcumin‐loaded GNP. The findings of the study establish that genipin crosslinking of GNP significantly modulates its ability to entrap and release the curcumin as a payload. Additionally, genipin crosslinking also influences the in vitro delivery of curcumin‐loaded GNP into cells. Furthermore, these studies confirm that genipin crosslinking of GNP enhances the cytotoxicity of curcumin as a payload against lung cancer (A549) by promoting cellular uptake through caveolae‐mediated endocytosis mechanism. The future studies should be conducted employing in vivo models to corroborate the in vitro results in order to realize the real benefits of this promising drug delivery system.

## Funding

No funding was received for this manuscript.

## Conflicts of Interest

The authors declare no conflicts of interest.

## Supporting Information

Method M1: Mathematical fitting of in vitro release kinetic to the Korsmeyer–Peppas equation. Table S1: Volumetric ratio of antisolvent required for the nanoprecipitation of aqueous gelatin (15 mg/mL). Table S2: Effect of the concentration of gelatin on the colloidal parameters of GNP. Figure S1: Representative UV–VIS spectra of GNP, GN‐GNP1, GN‐GNP2, and GN‐GNP3 in water and following reaction with ninhydrin (2% w/v) in water:ethanol mixture (50/50, v/v) at 90°C for 15 min. Figure S2: The size distribution function of GNP, GN‐GNP1, GN‐GNP2, and GN‐GNP3 in water as obtained from DLS studies. Figure S3: The plot shows the mean fluorescence intensity (MFI) of cells under various experimental conditions from a representative microscopic field (magnification 20x). The MFI was calculated by measuring the pixel intensity of a minimum of 25 cells from the microscopic field using ImageJ software. Figure S4: The plot shows the % viability of A549 cells following treatment with various vehicles (0.15% DMSO, GNP, GNP1, GNP2, and GNP3) for 48 h prior to MTT assay.

## Supporting information


**Supporting Information** Additional supporting information can be found online in the Supporting Information section.

## Data Availability

The data that support the findings of this study are available within the article or its Supporting Information.
