# Supplementary material for: Effect of Genipin Crosslinking on the Cellular Delivery of Gelatin Nanocarriers Using Curcumin as a Payload
Source: Int J Biomater. 2026 Feb 13;2026:9512576. doi: 10.1155/ijbm/9512576 (PMC12905463; doi:10.1155/ijbm/9512576)
Supplement: Supplementary file 1 — Supporting Information Additional supporting information can be found online in the Supporting Information section. [file IJBM-2026-9512576-s001.docx]

**Effect of genipin-crosslinking on the cellular delivery of gelatin nanocarriers using curcumin as a payload**

Ram Pada Das^1,2^, Minati Nayak^1,2^, Beena Gobind Singh^1,2^, Koushik Majee^2^, Amit Kunwar^1,2*^

^1^Homi Bhabha National Institute, Anushaktinagar, Mumbai – 400094, India

^2^Radiation and Photochemistry Division, Bhabha Atomic Research Centre, Mumbai-400085, India

* Correspondence Address: Dr. Amit Kunwar ([kamit@barc.gov.in](file:///E:\OLD%20C%20DRIVE%20(KBS%20PC%20Amit)\Desktop\Vishwa%20Radiation%20paper\DSePA%20patent\DSePA%20sicentific%20report\DSePA%20metabolism%20paper\CBI\DSePA%20metabolism%20paper\CBI\DSePA%20metabolism%20paper\kamit@barc.gov.in))

**Supplementary methods**

**M1. Mathematical fitting of *in vitro* release kinetic to Korsmeyer-Peppas equation**

Korsmeyer-Peppas model is commonly used to analyse complex drug release kinetics which may involve both Fickian and non-Fickian mechanisms [1]. According to this model, the first 60% of the drug release data is used to fit an exponential equation (1) with a varying release exponent value:

$\frac{C_{t}}{C_{\infty}}=kt^{n}$ (1)

Here,$C_{t}$/$C_{\infty}$ represents the fraction of curcumin released at a given time (*t*). The other parameters like *k* and *n* represent the release rate constate and release exponent respectively. As per this model, the value of *n* indicates the type of release mechanism. For instance, n ≤ 0.45 suggests the release of entrapped drug through Fickian mechanism generally due to the diffusion of release medium in to nanocarrier [1]. Whereas, n > 0.45 is indicative of non-Fickian mechanism involving both diffusion of release medium in to nanocarriers as well as relaxation of nanocarrier for the release of entrapped drug. Thus, to understand the mechanism of the release of curcumin from various nanocarriers (viz., GNP, GN-GNP1, GN-GNP2 and GN-GNP3), the release kinetics (percent release versus time) data corresponding to first ~60% of curcumin release was subjected to nonlinear fitting to equation (1) using Origin software (version 6.1). This analysis gave the exact relationship between % release and time in the form of Y=B*X^A, where Y = C_t_/C_∞_, B = rate constant (*k*), X = t and A = release exponent (*n*). The values of *k* and *n* determined by this analysis are listed below:

| **Nanocarriers** | ***k* value (h^-1^)** | ***n* value** |
| --- | --- | --- |
| GNP | 11.69 | 0.37 |
| GN-GNP1 | 10.78 | 0.42 |
| GN-GNP2 | 7.28 | 0.54 |
| GN-GNP3 | 6.92 | 0.58 |

**References:**

1) Das RP, Gandhi VV, Singh BG, Kunwar A. Balancing loading, cellular uptake, and toxicity of gelatin-pluronic nanocomposite for drug delivery: Influence of HLB of pluronic. J Biomed Mater Res A. 2022;110(2):304-315. doi: 10.1002/jbm.a.37287.

**List of supplementary tables**

**Table S1**. Volumetric ratio of antisolvent required for the nanoprecipitation of aqueous gelatin (15 mg/ml)

| **Acetone : aqueous gelatin (V/V)** | **Turbidity** | **Ethanol : aqueous gelatin (V/V)** | **Turbidity** |
| --- | --- | --- | --- |
| 1:1 | Seen | 1:1 | Not seen |
|  |  | 1:2.5 | Not seen |
|  |  | 1:5 | Seen |

**Table S2.** Effect of the concentration of gelatin on the colloidal parameters of GNP

| **Gelatin concentration (mg/ml)** | **Hydrodynamic size (nm)** | **Scattering counts (kcps)** | **PI** | **Zeta potential (mV)** |
| --- | --- | --- | --- | --- |
| 7.5 mg/ml | No particle |  |  |  |
| 15 mg/ml | 150 ± 8 | 24.3 | 0.13 | -9.45 |
| 30 mg/ml | 292 ± 17 | 31.6 | 0.13 | -8.56 |
| 50 mg/ml | 786 ± 83 | 86.5 | 0.06 | -8.12 |

**List of** **supplementary figures**

**Fig. S1**. Representative UV-vis spectra of GNP, GN-GNP1, GN-GPN2 and GN-GPN3 in water and following reaction with ninhydrin (2% w/v) in water : ethanol mixture (50/50, v/v) at 90 °C for 15 min. The arrow indicates scattering due to aggregation of nanoparticles. NIH – ninhydrin.

**Fig. S2.** The size distribution function of GNP, GN-GNP1, GN-GPN2 and GN-GPN3 in water as obtained from DLS studies.

**Fig. S3**. The plot shows the mean fluorescence intensity (MFI) of cells under various experimental conditions from a representative microscopic field (magnification 20X). The MFI was calculated by measuring the pixel intensity of a minimum of 25 cells from the microscopic field using image J software. Briefly, the cells were pretreated with inhibitors (Dyn - Dynasore; Fil – Filipin; Chl – Chlorpromazine) for 2 h followed by treatment with GN-GNP3-CUR at a curcumin equivalent concentration of 25 μM for 6 h and then processed for fluorescence imaging. The fluorescence images were captured after excitation using FITC filter equipped with microscope (Olympus IX83). The results are presented as mean ± SD (n = 25). The control represents untreated cells. The GN-GNP3 group represents cells treated with equivalent volume of blank nanocarrier without curcumin. The DMSO-CUR group represents cells treated with curcumin (25 μM) through DMSO (0.15%).; CUR – curcumin. ^*^ *p* < 0.05 as compared to control; ^$^ *p* < 0.05 as compared to GN-GNP3; ^#^ *p* < 0.05 as compared to DMSO-CUR; and ^@^ *p* < 0.05 as compared to GN-GNP-3-CUR. CUR – Curcumin.

**Fig. S4**. The plot shows the % viability of A549 cells following treatment with various vehicles (0.15% DMSO, GNP, GNP1, GNP2 and GNP3) for 48 h prior to MTT assay. The results are presented as mean ± SD (n =3). The difference in the means of various treatment groups are statistically insignificant (*p* > 0.05).
